# An Account of Diseases in an Eastern District of London, from December 20, 1808, to February 20, 1809

**Published:** 1809-03

**Authors:** 


### *An Account of Diseases in an Eastern District of London, from December* 20, 1808, *to February* 20, 1809.

| ACUTE DISEASES. | |
|---|---|
| Typhus - - - - - - - | 3 |
| Scarlatina - - - - - - | 2 |
| Peripneumonia Notha | 15 |
| Cynanche Tonsillaris - | 2 |
| Rheumatismus Acutus | 3 |

| CHRONIC DISEASES. | |
|---|---|
| Tussis - - - | 30 |
| Tussis cum Dyspnœa | 23 |
| Pertussis - - - - - - | 3 |
| Phthisis Pulmonalis - | 4 |
| Hydrothorax - - - - - | 4 |
| Hemiplegia - - - - - | 2 |
| Vertigo - - - - - - | 2 |
| Cephalalgia - - - - | 4 |
| Calculus - - - - - - | 1 |
| Gastrodynia - - - - - | 4 |
| Hypochondriasis - - | 4 |
| Dyspepsia - - - - - - | 6 |
| Vomitus - - - - | 3 |
| Dysmenorrhœa - - - | 3 |
| Menorrhagia - - - - | 5 |
| Fluor Albus - - - - | 7 |
| Rheumatismus Chronicus | 20 |
| Dysuria - - - - - - | 5 |

| PUERPERAL DISEASES. | |
|---|---|
| Convulsio - - - - - - | 1 |
| Menorrhagia Lochialis | 5 |
| Rhagas Papillæ - - - | 4 |
| Abscessus Mammæ - - | 2 |
| Enterodynia - - - - | 7 |
| Diarrhœa - - - - | 12 |

| INFANTILE DISEASES. | |
|---|---|
| Convulsio - - - - - - | 3 |
| Ophthalmia - - - - - | 5 |
| Vermes - - - - - - | 4 |
| Diarrhœa - - - - - - | 7 |

The uncommon severity of the weather, made it easy to anticipate what must be the diseases which would make the principal figure in the list. A continuation of those which were mentioned in the last Report, together with an aggravation of their symptoms, might well be expected. This is the season in which diseases that have their seat in the pulmonary system generally appear with the greatest frequency, and in the most threatening form. Coughs and Catarrhs, Peripneumonies and Pleurises, generally form a very large proportion of the diseases that come under the notice of the medical practitioner.

These being the common epidemic of the season, they may well be expected to prevail, in a considerable degree, at the present time, when the usual exciting cause has operated with an uncommon degree of violence. The immense quantity of snow that has fallen, and the constant succession of rain, have been the occasion of most alarming inundations in almost every part of the kingdom. These, besides the immediate mischief they have done in swelling torrents, tearing up bridges, overthrowing some buildings, and laying parts of others under water for a considerable time, have contributed to increase the number, and aggravate the symptoms, of human maladies.

The humid state of the atmosphere which has been hereby produced, has conspired in no small degree, with other other causes, to produce or protract those different complaints of the chest, which have lately been so prevalent.

Rheumatisms, of different species, have also occurred in a great number of instances; and in a disease, which is so much affected by frequent changes of temperature, it is not surprising that it should frequently recur at the present season in constitutions liable to its attack.

